# Adult attachment styles and the psychological response to infant bereavement

**DOI:** 10.3402/ejpt.v5.23295

**Published:** 2014-05-14

**Authors:** Mark Shevlin, David Boyda, Ask Elklit, Siobhan Murphy

**Affiliations:** 1 School of Psychology and Psychology Research Institute, University of Ulster at Magee, Londonderry, Northern Ireland; 2 National Centre of Psychotraumatology, University of Southern Denmark, Odense, Denmark

**Keywords:** Attachment typology, Bartholomew, latent profile analysis, depression, anxiety

## Abstract

**Background:**

Based on Bowlby's attachment theory, Bartholomew proposed a four-category attachment typology by which individuals judged themselves and adult relationships. This explanatory model has since been used to help explain the risk of psychiatric comorbidity.

**Objective:**

The current study aimed to identify attachment typologies based on Bartholomew's attachment styles in a sample of bereaved parents on dimensions of closeness/dependency and anxiety. In addition, it sought to assess the relationship between the resultant attachment typology with a range of psychological trauma variables.

**Method:**

The current study was based on a sample of 445 bereaved parents who had experienced either peri- or post-natal death of an infant. Adult attachment was assessed using the Revised Adult Attachment Scale (RAAS) while reaction to trauma was assessed using the Trauma Symptom Checklist (TSC). A latent profile analysis was conducted on scores from the RAAS closeness/dependency and anxiety subscales to ascertain if there were underlying homogeneous attachment classes. Emergent classes were used to determine if these were significantly different in terms of mean scores on TSC scales.

**Results:**

A four-class solution was considered the optimal based on fit statistics and interpretability of the results. Classes were labelled “Fearful,” “Preoccupied,” “Dismissing,” and “Secure.” Females were almost eight times more likely than males to be members of the fearful attachment class. This class evidenced the highest scores across all TSC scales while the secure class showed the lowest scores.

**Conclusions:**

The results are consistent with Bartholomew's four-category attachment styles with classes representing secure, fearful, preoccupied, and dismissing types. While the loss of an infant is a devastating experience for any parent, securely attached individuals showed the lowest levels of psychopathology compared to fearful, preoccupied, or dismissing attachment styles. This may suggest that a secure attachment style is protective against trauma-related psychological distress.

There has been a large corpus of literature that indicates that adult attachment is a powerful explanatory model for explaining psychopathology (Bifulco et al., 2006). The principle tenet of attachment theory proposes that the quality of early relationships is rooted in the degree to which an infant becomes reliant on the attachment figure as a source of security (Ainsworth, Blehar, Waters, & Wall, 1979). In times of distress, a normally functioning attachment system drives the infant towards their attachment figure for comfort. The caregiver facilitates this behaviour by being available, responsive, protective, and comforting when a threat or stressor presents itself (Waters, Kondo-Ikemura, Posada, & Richters, 1991). The availability, responsiveness, and active support of the caregiver provide the infant with the confidence to explore their environment, assured in the knowledge that the attachment figure is close by (Crowell & Treboux, 1995).

Bowlby (1969) argued that attachment relationships have a profound impact on the child's developing personality which is determined by the caregiver's emotional availability and responsiveness to the child's needs. Through continued interaction, a child's internal working model helps shape their beliefs about whether they are worthy of love and whether others can be trusted to provide love and support including the interpretation of interactions. Thus, another key principle of attachment theory is that early attachment relationships continue to be important throughout the life span; such experiences become the prototype or internal working model by which individuals judge later relationships. Ainsworth et al. (1979) identified three distinct patterns of infant attachment from observations of infants: secure, anxious–resistant, and avoidant. Securely attached infants seek proximity and welcome their caregivers’ return from absence, seeking comfort during times of distress. Anxious–resistant infants tend to show ambivalent behaviour towards caregivers and are resistant to the comforting behaviours of caregivers if distressed. Infants classified with an avoidant attachment style avoid proximity or interaction with the caretaker on reunion (Bartholomew & Horowitz, 1991).

Hazan and Shaver (1987) extended Bowlby and Ainsworth's work (Ainsworth et al., 1979; Bowlby, 1969) on infant–caregiver attachment to include adult romantic love which encapsulates three styles of behaviour termed, *secure*, *avoidant*, and *anxious* (or *anxious–ambivalent*). Hazan and Shaver (1987) found adults with a secure attachment style are comfortable depending on others and find it easy to get close to others. Adults with an avoidant attachment style are uncomfortable with close proximity with others and find trust an issue while adults with an anxious attachment style sees others as reluctant to get close to them and are often viewed as overly dependent on others (Mickelson, Kessler, & Shaver, 1997). There is some variability in the measures derived from Hazan and Shaver's (1987) original measure; however, several studies suggest that when the content of multiple-item measures is based closely on Hazan and Shaver’ (1987) descriptions, two major dimensions emerge: comfort with closeness and relationship anxiety (Feeney & Noller, 1996). Collins and Read (1990) later developed the Revised Adult Attachment Scale (RAAS) which is based on the earlier Adult Attachment Scale (AAS: Collins & Read, 1990) and on the work of Hazan and Shaver (1987). The newly revised scale included three discrete styles of attachment; closeness, anxiety, and dependency, which can be used to independently assess individuals on each of the three dimensions. Alternatively, it can be used to categorise individuals into four attachment styles by combining the dimensions of closeness and dependency. This results in a two-dimensional construct with four attachment styles, namely secure, preoccupied, dismissing, and fearful.

The original focus of attachment theory was parenting behaviour but has since been extended to help explain psychopathology (Dozier, 1990). In general, research has consistently found secure attachment style related to better mental health (Armour, Elklit, & Shevlin, 2011; Bunn, 2006). A number of studies using traumatised samples have found a negative relationship between secure attachment style and post-traumatic stress disorder (PTSD) (Dekel, Solomon, Ginzburg, & Neria, 2004; Fraley & Brumbaugh, 2004; O'Connor & Elklit, 2008). Among college students, poor parental marital quality (but not divorce) and parental drinking problems have been found to be related to insecure (dismissing) romantic attachment, especially avoidant attachment (Brennan & Shaver, 1993; Brennan, Shaver, & Tobey, 1991). Hazan and Shaver (1990) found that adults with insecure attachments report more depression and physical symptoms than those with secure attachments whereas Tasca et al. (2013) found that attachment insecurity mediated the relationship between childhood trauma and eating disorder psychopathology. Furthermore, Bakermans-Kranenburg and Van IJzendoorn (2009) reported that preoccupied attachment styles were associated with internalising behaviours (e.g., borderline personality disorders) while dismissing attachment style as associated with externalising behaviours (anti-social personality disorders). In line with previous literature, they also found associations between depressive symptomology and insecure attachment style.

The loss of a child has been reported to result in significantly higher levels of grief than the death of a partner or parent and often leads to complicated grief. Parents bereaved by infant death often exhibit a wide range of symptomatology, including depression, anger, anxiety, guilt, grief, and physical symptoms (Christiansen, Elklit, & Olff, 2013). In addition, research shows that infants born into a family with a history of child loss show higher rates of disorganised attachment to their mothers which may indicate that the aversive effects of child loss can have a profound influence on subsequent parent–child relationships (Cordle & Prettyman, 1994; Neugebauer et al., 1997; Thapar & Thapar, 1992). However, studies which have examined prenatal, perinatal, and post-natal losses have generally found no differences in symptomatology across types of loss (Dyregrov, 1990), although one study has reported more symptomatology following post-natal compared to prenatal loss (Gaudet, Séjourné, Camborieux, Rogers, & Chabrol, 2010).

There is significant psychological/psychiatric morbidity associated with infant loss (Blackmore et al., 2011); however, only a few studies have investigated attachment styles and psychological trauma in response to both peri- and post-natal mortality. The primary aim of the current study was to identify attachment styles, or “classes,” based on individuals’ relationship with their family members, romantic partners, and close friends. It was hypothesised that consistent with Bartholomew's (1990) findings, four distinct attachment styles would best represent the sample of bereaved parents. Second, demographic and infant-related loss variables were used as predictors of the attachment typology. Third, differences within the attachment styles on a broad range of psychological reactions to trauma were assessed. It was hypothesised that the severity of reaction to psychological trauma would vary as a function of attachment type, thus higher mean scores would be associated with fearful attachment style. The proposed three-step analysis is depicted in Fig. 1.

**Fig. 1:**
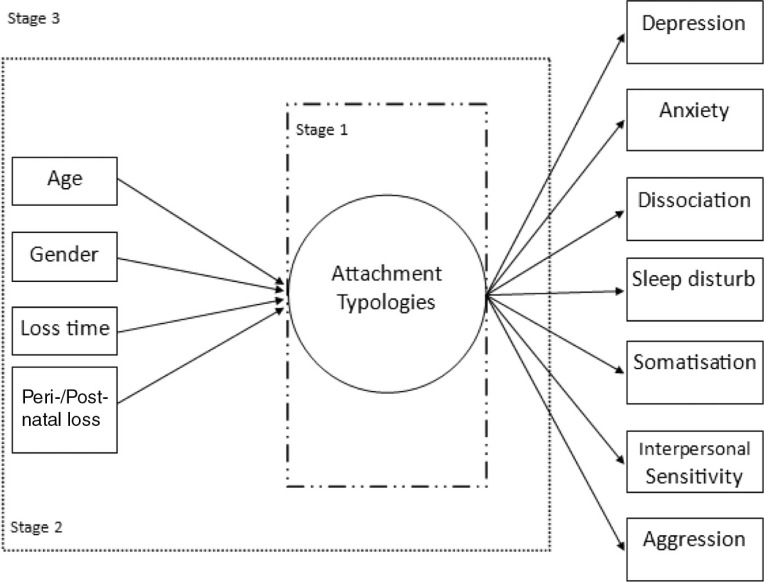
Model of the proposed three-stage analytic plan.

## Method

### Sample

Participants were all members of the Danish “National Association of Infant Death” which is a private organisation that provides counselling and support to bereaved parents and close family members. The association arranges support groups, annual services, memorials, and public lectures disseminating information and awareness of infant death. Bereaved individuals are referred to the association during their time in hospital or upon discharge. The Institutional Review Board approved the ethical standard of the study protocol. The study was also subject to a board decision within the National Association of Infant Death and granted permission to proceed.

Invitation letters were sent out together with the questionnaire. Participation was entirely voluntary and no reason for non-participation was requested. The information letters that detailed the study were sent out with a questionnaire pack to 650 households of members of the association. Each questionnaire pack included two copies of the survey; one for each parent and the response rate was (defined as a least one answer from one parent from one address) was 53%. The principal investigator's contact details were included in the invitation letter for further information or mental health needs for those members who agreed to participate.

The final sample consisted of 445 participants (254 females and 191 males) with an overall response rate of 68.4%. The inclusion criteria was that the time of loss had to occur within the past 5 years which was consistent with similar studies (Bennett, Litz, Maguen, & Ehrenreich, 2008; Murphy, Clark, & Lohan, 2002). The mean age of participants was 32.64 (SD=5.1). In this study, the term perinatal loss was used to describe a sample of parents who experienced loss from gestational week 18 until birth (*N*=242). The term post-natal loss was used to describe parents who lost their child within the first 2 years of life (*N*=199). The average time since the loss of the infant was 2.2 years (SD=1.2). There were missing data for four cases and they were therefore not included in the analysis.

## Measures

### The RAAS (Collins, 1996)

The RAAS is a measure of adult attachment based on the AAS (Collins & Read, 1990), which assesses close interpersonal relationships and is measured using a 5-point Likert scale (1=not at all characteristic) to (5=very characteristic of me). The RAAS consists of 18 items which measure three subscales: closeness, dependency, and anxiety. High scores on the anxiety dimension characterise individuals who worry about being unloved or abandoned by romantic partners. High scores on the closeness dimension characterise individuals who find closeness with others easy and high scores on the dependent dimension characterise individuals who feel that others are trustworthy and dependable (Collins, 1996). To test Bartholomew's (1990) four attachment styles (secure, preoccupied, fearful, and dismissing), closeness and dependency are combined into a composite scale resulting in two subscales: closeness/dependency and anxiety. The internal consistency of the subscales has been evidenced using clinical and non-clinical samples with Cronbach's α of 0.77 for closeness, 0.78 for dependency, and 0.85 for anxiety (Collins, 1996; Eng, Heimberg, Hart, Schneier, & Liebowitz, 2001). In the present study, Cronbach's α was 0.83 for anxiety and 0.76 for closeness/dependency. Prior to analysis, the scores on the closeness/dependency and anxiety scales were standardised to produce variables with a mean of zero and a standard deviation of one.

### The Trauma Symptom Checklist

The TSC33 was used to measure trauma-specific and psychological symptoms associated with the loss of an infant. The Trauma Symptom Checklist (TSC; Briere & Runtz, 1989) contains 33 items with five symptom subscales, which are rated in relation to the previous month (*How often have you experienced each of the following in the last month*). In this study, two items (Do you feel angry or irritated? Do you feel low in energy?) were added to the original TSC33 (Elklit, 1990). The seven scales are depression, anxiety, dissociation, sleep disturbances, somatisation, interpersonal sensitivity, and aggression. The revised version of the TSC35 has undergone psychometric testing (Elklit 1990, 1994; Elklit & Brink, 2003). Each item is scored on a 4-point scale (0=“Never,” 3=“Often”). The TSC35 has undergone psychometric testing (Elklit, 1990; Elklit & Brink, 2003). Elklit (1990) found adequate estimates of reliability for each of the subscales: depression (*α*=0.89), anxiety (*α*=0.82), dissociation (*α*=0.84), sleep disturbances (*α*= 0.87), somatisation (*α*=0.84), interpersonal sensitivity (*α*=0.74), and aggression (*α*=0.68). The overall reliability for the TSC was (*α*=0.95). The TSC has been credited as a valid measurement of the sequelae of traumatisation.

### Analytic plan

For the first step of the present study, a number of latent profile analysis (LPA) models were estimated (two classes to six classes) using Mplus 6 (Muthén & Muthén, 2010). LPA is a statistical method used to identify homogeneous groups, or classes, from continuous multivariate data and offers many advantages over traditional cluster analysis techniques (Pastor, Barron, Miller, & Davis, 2007). The LPA was based on scores from the closeness/dependency and anxiety subscales of the RAAS. Models were compared to determine the solution that provided the best fit to the data. Overall, model fit was determined by the Lo–Mendell–Rubin Test (LMRT: Lo, Mendell, & Rubin, 2001), an inferential statistical test that compares a target class solution (e.g., 2-class) to a class solution with one class less (e.g., 1-class). A LMRT probability value <0.05 indicates that the particular solution fits the data significantly better than the solution with one class less. When the LMRT value becomes non-significant (>0.05), it is an indication that a solution with one class less should be considered the best. The Akaike Information Criterion (AIC; Akaike, 1987), the Bayesian Information Criterion (BIC; Schwarz, 1978), and the sample size-adjusted BIC (ssaBIC; Sclove, 1987) were also assessed to determine model fit with lower values indicating better model fit. Entropy values indicate the ability of the model to correctly classify participants with high values indicating better classification (Ramaswamy, DeSarbo, Reibstein, & Robinson, 1993). Assessment of the AIC, BIC, and ssaBIC should be undertaken when determining the significance of additional classes. The second step of the study was to assess the relationship between demographic and peri- and post-natal variables with attachment typology. This was achieved by using a multinomial logistic regression (MNLR) where demographic and infant-related loss variables were used as predictors and the resultant LPA classes were included as the dependent variables. The third step of the analysis assessed a range of psychological trauma variables on the resultant classes using a test of mean differences.

## Results

### Latent profile analysis

The fit statistics are presented in Table 1. The 4-class solution was considered optimal due to the lower AIC and BIC values in comparison to classes 2 and 3 as well as producing a higher Entropy value than the 3-class solution. Classes 5 and 6 were rejected due the non-significant LRMT values. As a result, the 4-class solution was deemed the best-fitting model. Interpretation of the 4-class was as follows: Class 1 (fearful, 6.7%) reflected individuals who were high in anxiety and low on the closeness/dependency dimensions. Class 2 (dismissing, 4.3%) reflected individuals who were low on anxiety and lowest on closeness/dependency dimensions. Class 3 (secure, 64%) reflected individuals who had the lowest anxiety and highest scores on closeness/dependency. Class 4 (preoccupied, 25%) reflected individuals who had high anxiety but lower than the fearful class in addition to low scores on the closeness/dependency dimension despite the fact these were higher than the fearful and dismissing classes.

**Table 1:** Model fit indexes for the 2–6 class solutions

Model	Log likelihood	AIC	BIC	ssaBIC	Entropy	LMRT
2-class	1575.273	3172.546	3220.802	3185.880	0.84	193.307**
3-class	1530.138	3096.277	3175.241	3118.096	0.79	88.294**
4-class	1504.952	3059.904	3169.576	3090.209	0.82	49.271*
5-class	1481.966	3027.932	3168.312	3066.722	0.74	44.967
6-class	1469.746	3017.492	3188.581	3064.768	0.70	23.905

Note:

* =p<0.05

** =p<0.001

AIC=Akaike Information Criterion; BIC=Bayesian Information Criterion; ssaBIC=sample size-adjusted Bayesian Information Criterion; LMRT, Lo–Mendell–Rubin Test.

**Fig. 2:**
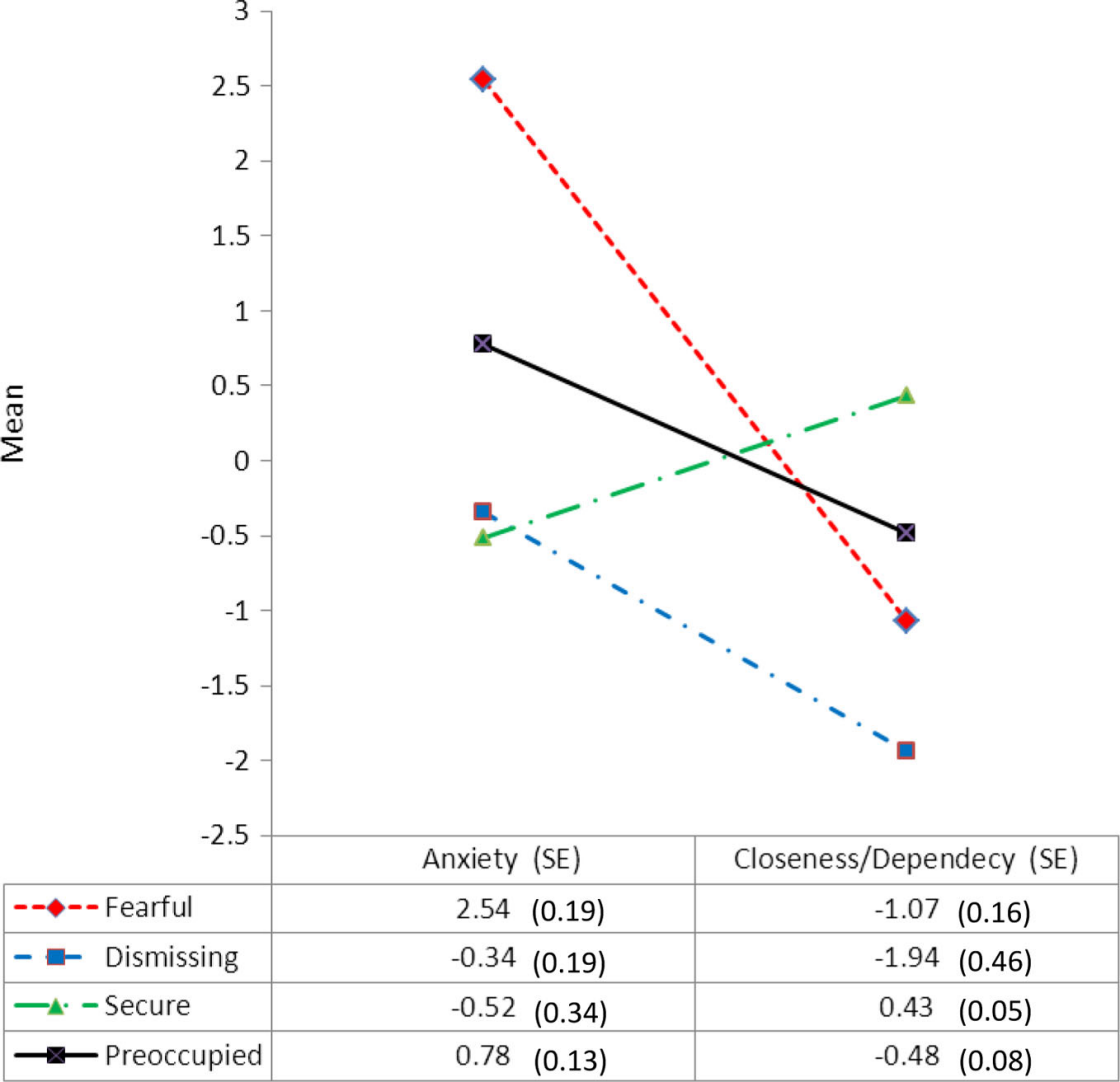
Four-class LPA plot with estimated means and probability scale (SE).

The latent profile plot is depicted in Fig. 2 with class means and standard errors.

The latent profile plot can be seen in Fig. 2 and shows both qualitative and quantitative differences between the four attachment styles with the fearful class lying diametrically opposite the secure class. The preoccupied attachment style lies centrally between the fearful and secure class. The dismissing class displays the lowest closeness/dependency mean scores; however, it exhibits marginally higher mean scores on anxiety over the secure class.

### Multinomial logistic regression

MNLR was used to assess the relationship between demographic and infant loss variables on the resultant classes, using the secure class as the comparison level. The odds ratio shows (Table 2) that females are significantly more likely than males to be in the fearful class compared to the secure class.

**Table 2:** Multinomial logistic regressions results for predictor variables predicting class membership

	Estimate	SE	Est./SE	*p*	Odds ratio
Fearful					
Gender	2.054	0.842	2.439	0.015	7.80
Age	−0.083	0.069	−1.209	0.227	0.92
Loss time	0.98	0.106	0.920	0.357	1.10
Peri-/post-natal	−0.407	0.402	−1.013	0.311	0.66
Dismissive					
Gender	−0.947	0.590	−1.605	0.108	0.38
Age	0.022	0.062	0.353	0.724	1.02
Loss time	−0.086	0.169	−0.511	0.610	1.02
Peri-/post- natal	−0.350	0.642	−0.545	0.586	0.70
Preoccupied					
Gender	−0.021	0.257	0.082	0.935	0.97
Age	0.002	0.025	0.092	0.926	1.00
Loss time	−0.006	0.050	0.127	0.899	0.99
Peri-post-natal	0.305	0.252	1.210	0.226	1.35

### Test of mean differences

A test of mean differences was used to ascertain the difference in mean scores on a broad range of psychological reactions to trauma (depression, anxiety, dissociation, sleep problems, somatisation, sexual interest, and aggression). Table 3 indicates that the fearful class exhibited the highest TSC subscale scores, and post-hoc tests indicated that these were significantly higher than all other classes. The secure and dismissive classes had the lowest or similar mean scores while the preoccupied class tended to be higher (but lower than the fearful class). In addition, an independent sample *t*-test was conducted to compare the peri-/post-scores on a total trauma score. There was no significant difference in trauma scores between the two groups of bereaved parents, *t*(587)=1.07, *p*>0.05.

**Table 3:** Mean scores and standard errors (SE) for TSC subscales across attachment classes

	Depression (SE)	Anxiety (SE)	Dissoc. (SE)	Sleep (SE)	Somat. (SE)	S. Intere. (SE)	Aggress. (SE)
Fearful	21.42 (0.83)**	14.03 (0.68)**	12.72 (0.62)**	7.06 (0.40)*	13.28 (0.65)**	13.56 (0.63)**	7.026 (0.30)**
Dismiss.	14.87 (0.78)	10.05 (0.66)	8.97 (0.49)	5.67 (0.45)	9.21 (0.13)	10.61 (0.64)	5.54 (0.33)
Secure	14.59 (0.18)	9.96 (0.12)	8.82 (0.11)	5.54 (0.10)	9.21 (0.65)	9.39 (0.11)	5.03 (0.06)
Preocc.	16.57 (0.36)	11.22 (0.26)	10.12 (0.26)	6.17 (0.20)	10.39 (0.26)	10.97 (0.26)	5.89 (0.14)
*χ* ^2^ *p*	90.616 0.000	54.546 0.000	60.283 0.000	73.936 0.000	54.396 0.000	77.068 0.000	73.603 0.000

Note:

* =p<0.05

** =p<0.001.

## Discussion

The primary aim of the current study was to identify attachment styles in a sample of bereaved parents. The results obtained were consistent with Bartholomew's (1990) four-dimensional model of attachment. Secure attachment constituted the largest class (64%) with the preoccupied group (25%) constituting the second largest class. Fearful (6.7%) and dismissing classes (4.3%) were the smallest classes. The sample distributions are comparable to those found in previous studies. For example, in a similar study which identified only three attachment styles, Armour et al. (2011) reported that the secure class (46.9%) encompassed the largest proportion of their sample whilst the preoccupied class (34.5%) represented the second largest with the fearful accounting for the smallest proportion (18.6%). In a nationally representative study, Mickelson, Kessler, and Shaver (1997) noted that the greatest proportion of the sample was characterised as having a secure attachment style.

The second aim was to identify predictors of class membership. The results showed that gender was the only significant predictor with females being almost eight times more likely than males to be in the fearful attachment class. These findings are comparable to the results from Reis and Grenyer (2004) who discovered that females who avoided intimacy for fear of rejection endorsed significantly higher levels of fearful attachment compared to males. In addition, they are in line with research by Waldinger, Schulz, Barsky, and Ahern (2006) who found that for females, fearful attachment fully mediated the link between childhood trauma and somatisation, whereas for men there was no such mediation. The results obtained from the final aim showed a significant difference in mean scores across a broad range of psychological responses to trauma as measured by the TSC. The fearful attachment style was significantly associated with higher scores on all measures of psychological trauma, particularly depression and anxiety compared to the secure attachment style which evidenced the lowest scores. This is strikingly similar to the results obtained by Reis and Grenyer (2004) who found significant relationships between fearful attachment and a broad spectrum of depressive symptoms. The dismissing and preoccupied styles also displayed lower scores than the fearful class but higher than the secure class on all outcome variables.

## Limitations

Several limitations to this study need to be acknowledged. First, this study is retrospective and cross-sectional; thus, speculation about causation between attachment typology and psychopathology cannot be extrapolated to the general population. Second, the use of self-report instruments may be problematic as biases could have overinflated or underinflated the relationship between measures. Third, individuals with attachment disturbances and psychopathology may have created biases (e.g., denial of symptoms), thereby precluding the accurate assessment of underlying symptomology. Fourth, although there is a large body of literature which has examined early childhood experiences and subsequent attachment styles, this study did not investigate the sampled early childhood experiences. That said, the theoretical consistency of the relation observed between attachment typology and pathology corroborate the findings of a great deal of the previous work in this field. These limitations notwithstanding, the current study includes a number of strengths which merit recognition. First, the sample involved a large group with high levels of interpersonal trauma and attachment insecurity alongside a reasonably balanced representation of males and females. Also, the use of LPA in the attachment research is more appropriate than traditional cluster analysis given that cluster analysis ascertains groupings based on observed homogeneity by determining the distance between cases. However, LPA determines groupings based on the response of the individuals, under the premise that individuals respond in a similar manner due to an overarching latent trait, in this case, an attachment style.

## Conclusions

In summary, the results of this study indicate four attachment styles within a bereaved sample of parents. The fearful and preoccupied attachment styles potentially pose the greatest risk factors for the development of psychiatric symptoms after the experience of trauma. The fearful class was most likely to be female and evidenced the highest scores on various psychopathology outcomes. Whilst the loss of an infant through pre- or post-natal complications can be a devastating loss for any parent, the results obtained from this study suggest that a secure attachment style may mitigate the effects of more serious psychiatric outcomes. It also potentially highlights the importance of assessing an individual's attachment style after serious psychological trauma as attachment difficulties may hinder the benefits of recovery in terms of therapeutic or medical intervention and subsequent social support.

## References

[CIT0001] Ainsworth M, Blehar M, Waters E, Wall S (1979). Patterns of attachment: A psychological study of the strange situation.

[CIT0002] Akaike H (1987). Factor analysis and AIC. Psychometrika.

[CIT0003] Armour C, Elklit A, Shevlin M (2011). Attachment typologies and posttraumatic stress disorder (PTSD), depression and anxiety: A latent profile analysis approach. European Journal of Psychotraumatology.

[CIT0004] Bakermans-Kranenurg M. J, Van IJzendoorn M. H (2009). The first 10,000 adult attachment interviews: Distributions of adult attachment representations in clinical and non-clinical groups. Attachment and Human Development.

[CIT0053] Bartholomew K (1990). Avoidance of Intimacy: An Attachment Perspective. Journal of Social and Personal Relationships.

[CIT0005] Bartholomew K, Horowitz L. M (1991). Attachment styles among young adults: A test of a four-category model. Journal of Personality and Social Psychology.

[CIT0006] Bennett S. M, Litz B. T, Maguen S, Ehrenreich J. T (2008). An exploratory study of the psychological impact and clinical care of perinatal loss. Journal of Loss and Trauma.

[CIT0007] Bifulco A, Kwon J, Jacobs C, Moran P. M, Bunn A, Beer N (2006). Adult attachment style as mediator between childhood neglect/abuse and adult depression and anxiety. Social Psychiatry and Psychiatric Epidemiology.

[CIT0008] Blackmore E. R, Côté-Arsenault D, Tang W, Glover V, Evans J, Golding  J (2011). Previous prenatal loss as a predictor of perinatal depression and anxiety. The British Journal of Psychiatry.

[CIT0009] Bowlby J (1969). Attachment and loss.

[CIT0010] Brennan K. A, Shaver P. R (1993). Attachment styles and parental divorce. Journal of Divorce and Remarriage.

[CIT0011] Brennan K. A, Shaver P. R, Tobey A. E (1991). Attachment styles, gender and parental problem drinking. Journal of Social and Personal Relationships.

[CIT0012] Briere J, Runtz M (1989). The Trauma Symptom Checklist (TSC-33) early data on a new scale. Journal of Interpersonal Violence.

[CIT0013] Bunn A (2006). Adult attachment style as mediator between childhood neglect/abuse and adult depression and anxiety. Social Psychiatry and Psychiatric Epidemiology.

[CIT0014] Christiansen D. M, Elklit A, Olff M (2013). Parents bereaved by infant death: PTSD symptoms up to 18 years after the loss. General Hospital Psychiatry.

[CIT0017] Collins N. L (1996). Working models of attachment: Implications for explanation, emotion and behavior. Journal of Personality and Social Psychology.

[CIT0018] Collins N. L, Read S. J (1990). Adult attachment, working models, and relationship quality in dating couples. Journal of Personality and Social Psychology.

[CIT0019] Cordle C. J, Prettyman R. J (1994). A 2-year follow-up of women who have experienced early miscarriage. Journal of Reproductive and Infant Psychology.

[CIT0020] Crowell J. A, Treboux D (1995). A review of adult attachment measures: Implications for theory and research. Social Development.

[CIT0021] Dekel R, Solomon Z, Ginzburg K, Neria Y (2004). Long-term adjustment among Israeli war veterans: The role of attachment style. Anxiety, Stress and Coping.

[CIT0023] Dozier M (1990). Attachment organization and treatment use for adults with serious psychopathological disorders. Development and Psychopathology.

[CIT0024] Dyregrov A (1990). Parental reactions to the loss of an infant child: A review. Scandinavian Journal of Psychology.

[CIT0025] Elklit A (1990). Måling af belastninger efter voldeligt overfald. Nordisk Psykologi.

[CIT0055] Elklit A (1994). Target drama at Aarhus University: an analysis of the psychological reactions and coping strategies. [The shooting drama at the University of Aarhus: An analysis of the psychological impact and coping strategies]. Psychological Skriftserie Aarhus.

[CIT0026] Elklit A, Brink O (2003). Acute stress disorder in physical assault victims visiting a Danish emergency ward. Violence and Victims.

[CIT0027] Eng W, Heimberg R. G, Hart T. A, Schneier F. R, Liebowitz M. R (2001). Attachment in individuals with social anxiety disorder: The relationship among adult attachment styles, social anxiety, and depression. Emotion.

[CIT0028] Feeney J. A, Noller P (1996). Adult attachment.

[CIT0030] Fraley C, Brumbaugh C. C, Rholes W. S, Simpson J. A (2004). A dynamical systems approach to conceptualizing and studying stability and change in attachment security. Adult attachment: Theory, research, and clinical implications.

[CIT0031] Gaudet C, Séjourné N, Camborieux L, Rogers R, Chabrol H (2010). Pregnancy after perinatal loss: Association of grief, anxiety and attachment. Journal of Reproductive and Infant Psychology.

[CIT0032] Hazan C, Shaver P (1987). Romantic love conceptualized as an attachment process. Journal of Personality and Social Psychology.

[CIT0033] Hazan C, Shaver P. R (1990). Love and work: An attachment-theoretical perspective. Journal of Personality and Social Psychology.

[CIT0035] Lo Y, Mendell N. R, Rubin D. B (2001). Testing the number of components in a normal mixture. Biometrika.

[CIT0036] Neugebauer R, Kline J, Shrout P, Skodol A, O'Connor P, Geller P. A (1997). Major depressive disorder in the 6 months after miscarriage. JAMA.

[CIT0037] Mickelson K. D, Kessler R. C, Shaver P. R (1997). Adult attachment in a nationally representative sample. Journal of Personality and Social Psychology.

[CIT0040] Murphy S, Clark L, Lohan J (2002). The aftermath of the violent death of a child: An integration of the assessments of parents’ mental distress and PTSD during the first 5 years of bereavement. Journal of Loss and Trauma.

[CIT0041] Muthén L. K, Muthén B. O (2010). Mplus user's guide.

[CIT0042] O'Connor M, Elklit A (2008). Attachment styles, traumatic events, and PTSD: A cross-sectional investigation of adult attachment and trauma. Attachment and Human Development.

[CIT0054] Pastor D. A, Barron K. E, Miller B. J, Davis S. L (2007). A latent profile analysis of college students' achievement goal orientation profiles. Contemporary Educational Psychology.

[CIT0043] Ramaswamy V, DeSarbo W. S, Reibstein D. J, Robinson W. T (1993). An empirical pooling approach for estimating marketing mix elasticities with PIMS data. Marketing Science.

[CIT0044] Reis S, Grenyer B. F. S (2004). Fear of intimacy in women: Relationship between attachment styles and depressive symptoms. Psychopathology.

[CIT0046] Schwarz G (1978). Estimating the dimension of a model. The Annals of Statistics.

[CIT0047] Sclove S. L (1987). Application of model-selection criteria to some problems in multivariate analysis. Psychometrika.

[CIT0056] Tasca G. A, Ritchie K, Zachariades F, Proulx G, Trinneer A, Balfour  L (2013). Attachment insecurity mediates the relationship between childhood trauma and eating disorder psychopathology in a clinical sample: a structural equation model. Child Abuse & Neglect.

[CIT0050] Thapar A. K, Thapar A (1992). Psychological sequelae of miscarriage: A controlled study using the general health questionnaire and the hospital anxiety and depression scale. The British Journal of General Practice.

[CIT0051] Waldinger R. J, Schulz M. S, Barsky A. J, Ahern D. K (2006). Mapping the road from childhood trauma to adult somatization: The role of attachment. Psychosomatic Medicine.

[CIT0052] Waters E, Kondo-Ikemura K, Posada G, Richters J. E, Gunner M, Sroufe A (1991). Learning to love: Milestones and Mechanisms. Minnesota symposium on child psychology.

